# Mobile Health Interventions: A Frontier for Mitigating the Global Burden of Cardiovascular Disease

**DOI:** 10.7759/cureus.62157

**Published:** 2024-06-11

**Authors:** Maryyam Liaqat, Maham Mushtaq, Ahmed Jamil, Muhammad Muaz Mushtaq, Husnain Ali, Rahma Anwar, Ahmad Raza, Asma Aslam, Tamseer Tariq, Muzaffer Hussain, Danyal Bakht, Syed Faqeer Hussain Bokhari

**Affiliations:** 1 Internal Medicine, King Edward Medical University, Lahore, PAK; 2 Medicine and Surgery, King Edward Medical University, Lahore, PAK; 3 Internal Medicine, Mayo Hospital, Lahore, PAK; 4 Medicine and Surgery, Karachi Medical and Dental College, Karachi, PAK; 5 Surgery, Karachi Medical and Dental College, Karachi, PAK; 6 Medicine and Surgery, Mayo Hospital, Lahore, PAK; 7 Surgery, King Edward Medical University, Lahore, PAK

**Keywords:** public health, healthcare, artificial intelligence, review, coronary artery disease, mhealth, cardiovascular

## Abstract

Mobile health (mHealth) interventions have emerged as a promising approach for cardiovascular disease (CVD) prevention and management. The proliferation of smartphones and wearable devices enables convenient access to health monitoring tools, educational resources, and communication with healthcare providers. mHealth interventions encompass mobile apps, wearables, and telehealth services that empower users to monitor vital signs, adhere to medication, and adopt healthier lifestyles. Their effectiveness hinges on user engagement, leveraging behavioral science principles and gamification strategies. While mHealth offers advantages such as personalized support and increased reach, it faces challenges pertaining to data privacy, security concerns, and resistance from healthcare providers. Robust encryption and adherence to regulations like the Health Insurance Portability and Accountability Act (HIPAA) are crucial for safeguarding sensitive health data. Integrating mHealth into clinical workflows can enhance healthcare delivery, but organizational adjustments are necessary. The future of mHealth is closely intertwined with artificial intelligence (AI), enabling remote monitoring, predictive algorithms, and data-driven insights. Tech giants are incorporating advanced health-tracking capabilities into their devices, paving the way for personalized wellness approaches. However, mHealth grapples with ethical dilemmas surrounding data ownership, privacy breaches, and inadvertent data capture. Despite its potential, mHealth necessitates a concerted effort to overcome obstacles and ensure ethical, secure, and practical implementation. Addressing technical challenges, fostering standardization, and promoting equitable access are pivotal for unlocking the transformative impact of mHealth on cardiovascular health and reducing the global burden of CVD.

## Introduction and background

Cardiovascular disease (CVD) presents a substantial worldwide health challenge, responsible for 31% of all mortality, with coronary heart disease (CHD) as a predominant contributor. Nevertheless, the World Health Organization (WHO) estimates that over 75% of premature CVD is avoidable, underscoring the importance of risk reduction [1]. Age, gender, educational level, and lifestyle practices are linked to CVD, highlighting the need for interventions, especially in lower- and middle-income countries (LMICs) [2]. Numerous population-based studies from LMICs have established the association between socio-demographic factors and CVD prevalence. Factors like age, gender, lower educational attainment, and behaviors such as smoking, alcohol consumption, physical inactivity, and poor dietary choices are linked to heightened CVD risk. Hypertension is notably a significant risk factor. Understanding these risk elements provides an opportunity for developing interventions aimed at diminishing CVD burdens in aging populations [3-5].

The digital era has introduced innovative strategies for addressing CVD. Mobile health (mHealth) has emerged as a potent tool for promoting health and managing diseases. Mobile phones, now ubiquitous, serve as versatile instruments for health education and the management of chronic diseases. With 93% of the global population having access to mobile-broadband networks, mHealth has vast potential to reach individuals, even in remote or underserved areas, enhancing access to health information [6]. Digital health, encompassing eHealth, mHealth, telehealth, health information technology, and telemedicine, is revolutionizing healthcare delivery [7]. It harnesses advanced computing, big data, genomics, and artificial intelligence (AI) to improve disease prevention, diagnosis, and management [8]. mHealth offers numerous opportunities for preventing CVD. Mobile applications and digital platforms can be customized for education, lifestyle tracking, and real-time vital sign monitoring. They empower individuals to manage their health by making informed decisions and adhering to risk reduction strategies [9].

This comprehensive narrative review delves into the dynamic synergy between mHealth interventions and CVD prevention, addressing a prominent global health concern. With the increasing prevalence of smartphones and wearable devices, mHealth interventions hold promise for CVD prevention. The review thoroughly examines mHealth interventions, including their types, advantages, user engagement, data security, and integration into healthcare. It critically assesses their effectiveness, acknowledging their potential and limitations. Furthermore, the review explores future trends, ethical considerations, and the pressing need for health equity. This review elucidates mHealth's role in safeguarding cardiovascular health and alleviating the CVD burden in our increasingly digital world.

## Review

Risk factors and prevalence

CVD comprises a broad spectrum of conditions affecting the cardiovascular system. Although CVD can have various etiological origins, such as embolic events leading to ischemic strokes in patients with atrial fibrillation or rheumatic fever contributing to valvular heart disease, addressing risk factors associated with atherosclerosis is of paramount importance, given its pivotal role in the pathogenesis of CVD [10]. Prominent risk factors for CVD have been established through extensive research. These include elevated levels of low-density lipoprotein (LDL) and cholesterol, tobacco use, hypertension, diabetes, abdominal adiposity, psychosocial factors, inadequate consumption of fruits and vegetables, excessive alcohol intake, and insufficient physical activity [11]. On a global scale, CVD holds the dubious distinction of being the foremost cause of mortality. As per the WHO, in 2015, approximately 17.7 million individuals succumbed to CVD. In the United States, CVD has consistently ranked as one of the top two leading causes of death since 1975, accounting for 25% of all deaths. In 2015, heart disease claimed 633,842 American lives, emerging as the principal cause of death, followed by cancer [10]. The global prevalence of CVD has displayed a worrisome upward trajectory, with the number of cases nearly doubling from 1990 to 2019. Concurrently, CVD-related mortality rates have significantly risen during this period [12]. This public health challenge is further compounded by its substantial economic burden on society. The indirect costs associated with CVD are estimated at $237 billion annually, with projections indicating a surge to $368 billion by 2035 [13].

Despite advances in diagnostic modalities and therapeutic interventions, the risk of heart disease remains considerable, with an estimated 50% risk by age 45 in the general population [10]. This risk escalates with advancing age and exhibits gender-specific variations, being higher in males at younger ages but progressively converging in the postmenopausal period [14]. Hence, addressing risk factors and advocating heart-healthy lifestyles early in life are imperative.

mHealth interventions

Mobile health, commonly known as mHealth, represents a groundbreaking force within the healthcare domain. The WHO defines mHealth as the practice of medical and public health supported by mobile devices, encompassing mobile phones, patient monitoring tools, personal digital assistants, and various wireless devices [15]. This pioneering approach is reshaping healthcare delivery and accessibility, potentially revolutionizing the conventional healthcare landscape [16]. In a world where mobile phone ownership exceeds 80% of the American population and smartphone usage is projected to surpass 80% in the next decade, mHealth possesses an extensive and expanding platform [17]. This is particularly critical as the incidence of chronic medical conditions continues to surge among American adults, resulting in suboptimal medical outcomes and escalated healthcare expenditures. Chronic conditions affect individuals across all age groups, underscoring the necessity for innovative solutions [18,19].

The elegance of mHealth lies in its simplicity. Basic mHealth features encompass voice communication and text messaging, both of which can be executed using cost-effective mobile phones. These methods have been harnessed in various healthcare applications, ranging from supporting cardiac rehabilitation programs to facilitating communication between consumers and clinicians in disease management [17]. mHealth systems operate similarly to traditional telehealth applications, yet with the added convenience of mobile applications on smartphones or tablets. Even in regions with unreliable cellular or wireless connectivity, data can be stored and transmitted once the connection is reestablished, ensuring healthcare continuity without interruptions stemming from connectivity issues [17]. This feature renders mHealth particularly valuable in cases where adherence to schedules and interventions is paramount, leading to enhanced health outcomes, especially in managing conditions like hypertension [17,20].

Moreover, mHealth interventions extend beyond patient care, aiming to enhance healthcare service delivery processes and offering comprehensive support and services to healthcare providers. These encompass education, diagnostic aid, and patient management [21]. Additionally, mHealth streamlines communication between healthcare services and consumers by dispatching appointment reminders and test result notifications, effectively dismantling geographical, temporal, and other barriers [21,22].

mHealth and CVD

CVD continue to present a significant global health challenge, emphasizing the crucial role of early detection and prevention in mitigating associated risks and healthcare expenditures. The advent of mHealth has heralded a new era of accessible healthcare solutions. Mobile applications equipped with heart rate monitoring functionality have emerged as a valuable asset in promoting cardiovascular health. These applications, readily accessible on smartphones, provide an economical and convenient avenue for heart rate monitoring, rendering them indispensable for individuals seeking to enhance their health and fitness. Whether employed for tracking exercise routines at the gym or monitoring daily activities, these applications instill confidence in users regarding their cardiovascular well-being [23].

The assimilation of information and communication technology into healthcare systems, notably through mHealth interventions, has transformed cardiac rehabilitation services. These interventions furnish evidence-based guidance in an engaging and user-friendly format, leading to reduced healthcare expenditures [24]. Research underscores the capacity of telehealth to effectively substitute or complement traditional cardiac rehabilitation, resulting in better management of cardiovascular risk factors and reduced clinical events [25]. This innovation holds immense significance in perpetuating adherence to a healthy lifestyle over the long term.

A plethora of mHealth apps offer an array of features, encompassing workout tracking, medication reminders, and general health monitoring facilitated by smartphone sensors. Some applications also offer manual data entry options, including meal tracking and weight loss guidance [26]. These diverse functionalities empower individuals to tailor their cardiovascular health management in accordance with their specific requirements and preferences. The widespread adoption of smartphones and tablet devices has unlocked the potential for ubiquitous health services. With their extensive processing capabilities, sensory functions, and communication features, these devices now serve as the primary technology for delivering mHealth solutions, rendering healthcare more accessible than ever [27].

mHealth offers distinct advantages compared to traditional secondary prevention programs, including flexible intervention delivery, personalized support, cultural sensitivity, increased reach, and reduced socioeconomic disparities in accessibility [28]. Furthermore, research underscores a growing trend where individuals with or at risk of CVD are increasingly embracing wearables for health monitoring. This tendency is particularly prominent in men. Users are inclined to share their health data with healthcare providers. The potential for clinically validated mHealth interventions to reach a broader demographic for CVD prevention and management is evident [29].

Components of mHealth interventions

The assimilation of mobile medical applications, wearable technology, and telemedicine has engendered substantial alterations in the approach to medical care and healthcare services by clinicians, patients, and healthcare providers. The widespread adoption of smartphones and tablets has led to a significant increase in the use of mobile medical applications, which play a crucial role in streamlining various medical tasks. These apps provide healthcare professionals with a range of tools for clinical reference, drug dosage calculation, patient record retrieval, and clinical decision support, benefiting various medical specialties and treatment approaches. They offer unprecedented access to medical information and patient data, enhancing the delivery of informed and effective healthcare for both practitioners and patients alike [30].

Wearable technology has also emerged as a focal point of global research attention. Propelled by rapid advancements in the internet, intelligent hardware, and big data, wearable devices have become integral to everyday life. These devices, including smartwatches, smart bracelets, armbands, and eyewear, have extended their reach into the healthcare domain [31]. They manifest as portable medical and health electronic gadgets worn directly on the body. Wearables possess the ability to perceive, record, scrutinize, and regulate various health parameters, potentially contributing to disease management [32]. Wearables facilitate the immediate detection of patient vital signs and laboratory metrics by seamlessly integrating mechanical functionalities with microelectronics and computational capabilities. They also offer functionalities like exercise guidance and medication reminders. The overarching objective is to deliver real-time, precise, and intelligent physiological and pathological data analysis, thereby facilitating self-diagnosis and self-monitoring [33].

In parallel, telemedicine has emerged as a highly advantageous technology that surmounts barriers to healthcare accessibility. It acts as a lifeline, particularly for those facing financial or geographical obstacles to quality healthcare. Telehealth enhances healthcare effectiveness, organization, and availability. Patients can now receive medical attention at their convenience, while healthcare professionals can deliver care securely and efficiently. The potential dividends of telemedicine are extensive, heralding a future in which healthcare is not only accessible but also optimized for the benefit of both patients and healthcare providers [34].

Effectiveness of mHealth interventions

In recent years, mHealth technology has emerged as a promising instrument in preventing and managing CVD. A multitude of investigations have documented the efficacy of mHealth-based interventions in individuals with CVD [35]. Chow et al.'s study demonstrated that the implementation of lifestyle-focused text messaging led to a noteworthy improvement in LDL-C levels in patients diagnosed with CHD [36]. Furthermore, web-based telemonitoring systems have proven efficacious for patients discharged with acute coronary syndrome [37]. The outcomes of studies aiming to manage blood pressure through mHealth tools have shown varying results, with a meta-analysis indicating a notable reduction in systolic blood pressure [38].

mHealth technology positively impacts various facets of cardiovascular health, including enhancements in exercise capacity, physical performance, medication adherence, quality of life (both physical and mental), and reduced hospital readmissions for both cardiovascular and non-cardiovascular causes [24]. Lifestyle-focused SMS text messages are effective in promoting self-management of a healthy diet, exercise, and medication adherence [39]. The ability to motivate and augment physical activity through mHealth tools contributes to tangible enhancements in cardiovascular health [40].

Nonetheless, despite the promising potential, mHealth confronts substantial challenges and limitations. Notably, many mHealth interventions lack a robust theoretical foundation. Some smartphone apps lack behavior change techniques (BCTs), which are imperative for sustained effectiveness. Furthermore, the regulatory framework governing medical apps remains a point of concern. Another notable challenge is the potential digital divide, which refers to unequal access to digital technology and the internet. Although internet and mobile phone access is on the rise, disparities persist and necessitate attention for mHealth interventions to achieve their full potential [28].

User engagement and behavior

In the domain of digital healthcare, mHealth programs have assumed a prominent role, affording individuals the capacity to self-manage their health. The efficacy of these programs, primarily directed toward fostering behavioral modifications and enhancing health, hinges upon user engagement [41]. User engagement transcends the mere attraction of users; it encompasses the sustenance of their participation over an extended duration [42]. Subpar engagement may erode the potential impact of mHealth, as users may not fully realize the benefits. To enhance engagement, two pivotal steps are imperative. Firstly, content development necessitates adherence to established health intervention guidelines, ensuring alignment with empirically proven effectiveness. Secondly, formulating the intervention's delivery modality, encompassing aspects such as navigation and visual elements, engenders a favorable user experience that distinguishes it from competitors, thus fostering engagement [43].

Behavioral science assumes a pivotal role in the crafting of efficacious mHealth interventions. The incorporation of theories pertaining to behavior change and the utilization of BCTs augments the likelihood of success. Behavioral design (BD) furnishes systematic methodologies for creating behavior change interventions. User-centric design processes resonate more effectively with users, thereby stimulating engagement and enhancing overall effectiveness [44].

Gamification is an emerging trend within mHealth to incentivize healthy behavioral transformations. Wearable tracking devices and mobile applications offer real-time feedback, serving as motivational tools for promoting physical activity [45,46]. Gamification, particularly when combined with social incentives, exhibits significant potential. While still evolving, empirical evidence concerning its effectiveness in inducing behavior change is progressively emerging. The application of gamification in mHealth portends the prospect of heightened user engagement and the cultivation of healthier behaviors [45].

Data security and privacy

Amid the remarkable benefits offered by mHealth, concerns pertaining to data privacy have surfaced as pivotal focal points. The sensitivity of personal health data collected via mHealth applications elicits valid apprehensions regarding privacy. Individuals frequently harbor concerns about the potential exposure of their health status to family, colleagues, or acquaintances, apprehensive of possible judgment, discrimination, or repercussions. The specter of social stigmatization intensifies the necessity for stringent privacy safeguards in mHealth applications [47]. User perspectives on interactions and communication significantly shape their decisions regarding data privacy, encompassing various issues such as regulating visibility in social networks and utilizing smartphone apps that gather sensitive information [48].

Implementing robust security protocols is imperative to assuage these privacy concerns. Encryption stands out as an efficacious and uncomplicated means to bolster data security. The utilization of WPA2 encryption on wireless devices holds the potential to significantly enhance the security of data conveyed across wireless networks. It is of paramount importance, however, to ensure the activation of this encryption on mobile devices. Additionally, it behooves researchers and developers to contemplate the integration of protocols for remote data wiping and device locking on mobile devices used for mHealth purposes. These systems, accessible across various operating systems or as supplementary features, facilitate remote data erasure and device lockdown in cases of loss or theft, thereby ensuring data safeguarding [49].

Within the United States, the Health Insurance Portability and Accountability Act (HIPAA), enacted in 1996, represented a seminal stride in addressing medical data privacy. HIPAA and analogous regulations, such as the General Data Protection Regulation (GDPR), accord primacy to individuals' rights concerning health information access, correction, trust, informed consent, and the necessity of data collection and utilization. Adherence to these regulations assumes critical importance for mHealth applications [50]. Providing a transparent, easily comprehensible privacy policy, encompassing user entitlements, data retention policies, and administrative particulars, assumes paramount significance. Users should be empowered to access their health data and be apprised of the repercussions of withholding it [51].

Healthcare provider involvement

Integrating mHealth into clinical practice is reshaping healthcare professionals' approach to patient care. Traditional healthcare predominantly relied on desktop computers, which constrained the accessibility of vital patient information. In contrast, mHealth technologies furnish healthcare professionals with real-time access to patient data, granting them enhanced mobility [52,53]. This enhanced mobility proves particularly advantageous in urgent medical situations, enabling rapid decision-making and continuous patient vital sign monitoring. The assimilation of mHealth solutions effectively addresses the challenges related to timely data retrieval and communication at the point of care. This seamless integration contributes to heightened job satisfaction and improved patient outcomes, ensuring the efficient delivery of healthcare services [52].

mHealth interventions usher in a new epoch of patient engagement, employing self-monitoring tools, reminders, motivational communications, and educational resources. These interventions empower patients by providing insights into their health data, allowing them to manage their well-being actively. Furthermore, mHealth facilitates communication between patients and healthcare providers, fostering collaborative interactions [54]. The integration of mHealth apps with wearables offers consumers even more potent tools to engage with their health data. Technological advancements in mHealth are reshaping the dynamics of communication between healthcare consumers and providers, transforming the delivery and reception of care, and enhancing access to health information [55].

However, despite the extensive potential of mHealth, some healthcare professionals underutilize mHealth applications. Resistance to electronic health technologies, such as mHealth, often originates from the need for organizational adjustments. Healthcare providers must adapt to these evolving technologies, which may encounter resistance on technical, individual, and organizational levels [56]. Additionally, privacy and data security concerns further complicate the adoption of mHealth. Users frequently express uncertainty about the data collected and stored by mHealth applications, as well as the entities with access to this data and its intended use. Security and privacy concerns are particularly pronounced for apps addressing sensitive issues like HIV/AIDS or mental health. Recent instances of data breaches underscore the critical significance of robust security measures within mHealth apps. Unfortunately, many of these apps lack sufficient security features and privacy policies, putting user data at risk [57].

Future trends and innovations

The landscape of mHealth is rapidly evolving, driven by advances in AI and technological innovations. Telehealth has become a primary method for noncritical patient care, making mHealth a crucial component of remote healthcare infrastructure. The adoption of mHealth devices has surged, facilitating the collection of extensive patient data, ideal for training AI models. Machine learning algorithms running on smart and wearable devices yield novel insights, supporting healthcare professionals in making more informed decisions. The synergy between AI and mHealth technologies is pivotal for advancing remote healthcare infrastructure [58]. AI-driven mHealth apps enable the remote monitoring of individuals with chronic diseases, enhancing their care and outcomes [59]. Recent developments have shown clinically significant improvements in morbidity and mortality outcomes in specific scenarios, underscoring mHealth's potential to enhance overall patient health [60]. The future of mHealth is closely tied to tech giants, which have integrated health activity tracking into their smartphones [61,62]. These innovations go beyond step counting, with companies expanding into activities like weightlifting and yoga. Smart clothing allows continuous data collection during workouts, providing more accurate measurements than wrist-based devices. Accelerometers and heart rate sensors are becoming more versatile, opening up new possibilities for predictive algorithms. Companies are facilitating cloud-based data aggregation and analysis to manage the wealth of information generated by these devices. Integrating this data with electronic medical record systems has the potential to transform cardiovascular health, offering personalized wellness approaches and valuable feedback to healthcare providers [63]. As people increasingly seek online information to prevent and treat CVD, harnessing infodemiology becomes pivotal in understanding how individuals access and engage with health-related information [64].

Challenges and ethical considerations

mHealth holds promise in addressing a spectrum of health issues. Nonetheless, it presents substantial challenges. Technical challenges are particularly salient. The presence of standardized healthcare practices helps communication among healthcare practitioners, developers, and researchers. Conversely, mHealth possesses the potential to expedite the assimilation of these standards, contingent on the willingness of entities and individuals to adopt them. The proliferation of extant standards, along with regional incongruities, can hinder endeavors to forge a cohesive mHealth framework. In privatized healthcare settings, the juxtaposition of service quality and financial considerations can prejudice patients [65].

Security emerges as a paramount preoccupation. Mobile applications, including mHealth apps, stand vulnerable to threats that could compromise the integrity and confidentiality of health-critical data. Safeguarding the security of mHealth apps assumes critical importance to preclude repercussions such as the falsification of clinical data, potentially resulting in unwarranted healthcare interventions and concomitant medicolegal and social implications. While mHealth harbors immense potential, a comprehensive approach is imperative to surmount these challenges, encompassing all facets of the healthcare milieu. The endeavor extends beyond patient empowerment, aiming to forge a secure, standardized, and cost-effective healthcare ecosystem where mHealth harmoniously interfaces with traditional healthcare paradigms. Application developers, healthcare stakeholders, and regulators collectively assume pivotal roles in shaping the future landscape of digital health.

In the realm of mHealth, ethical dilemmas emerge. Four key challenges stand out. Firstly, granting patients access to their raw data raises concerns. While it can empower patients, it also risks misinterpretation and unwarranted anxiety. Secondly, the issue of data ownership arises as patients share personal information with mHealth systems, necessitating a deeper exploration of potential co-ownership. Thirdly, privacy and security concerns are significant due to the portability of mHealth data, risking data breaches and compromised confidentiality. Lastly, inadvertent data capture of unconsenting individuals threatens privacy and autonomy. Addressing these ethical challenges is vital for shaping an ethical mHealth system [66].

## Conclusions

mHealth interventions have emerged as a promising strategy for tackling the global issue of preventing CVD. The increasing prevalence of smartphones and wearable devices has made mHealth a convenient and accessible way to promote cardiovascular well-being. These interventions encompass a range of tools, including mobile apps, wearable gadgets, and telehealth services, which empower users to monitor vital signs, adhere to medication regimens, and adopt healthier lifestyles. The effectiveness of mHealth relies heavily on user engagement, which integrates behavioral science principles and gamification. However, mHealth faces challenges concerning data security and privacy, along with resistance from healthcare providers. The future of mHealth is closely linked with AI and tech industry leaders, promising personalized healthcare and data-driven insights. In the realm of ethics, mHealth grapples with data ownership, privacy, and security issues. Addressing these challenges and promoting standardized practices will play a pivotal role in shaping the future of digital health. Overall, mHealth offers excellent potential to reduce the burden of CVD, but it demands a concerted effort to overcome obstacles and ensure ethical, secure, and practical implementation.
